# Relationship Between Heart Rate Variability and Pulse Rate Variability Measures in Patients After Coronary Artery Bypass Graft Surgery

**DOI:** 10.3389/fcvm.2021.749297

**Published:** 2021-12-16

**Authors:** Yung-Sheng Chen, Yi-Ying Lin, Chun-Che Shih, Cheng-Deng Kuo

**Affiliations:** ^1^Department of Exercise and Health Sciences, University of Taipei, Taipei, Taiwan; ^2^Tanyu Research Laboratory, Taipei, Taiwan; ^3^Institute of Emergency and Critical Care Medicine, National Yang-Ming-Chiao-Tung University, Taipei, Taiwan; ^4^Division of Cardiovascular Surgery, Department of Surgery, Taipei Municipal Wan Fang Hospital, Taipei, Taiwan; ^5^Department of Surgery, Taipei Medical University School of Medicine, Taipei, Taiwan; ^6^Department of Medical Research, Taipei Veterans General Hospital, Taipei, Taiwan; ^7^Research and Development Department VI, Smart Healthcare Business Unit (BU), Leadtek Research Inc., Taipei, Taiwan; ^8^Department of Medicine, Taian Hospital, Taipei, Taiwan

**Keywords:** autonomic nervous modulation, heart rate variability, pulse rate variability, coronary artery bypass graft, photoplethysmographic assessment

## Abstract

**Background:** Heart rate variability (HRV) and pulse rate variability (PRV) measures are two kinds of physiological indices that can be used to evaluate the autonomic nervous function of healthy subjects and patients with various kinds of illness.

**Purpose:** In this study, we compared the agreement and linear relationship between electrocardiographic signals (ECG)-derived HRV and photoplethysmographic signals (PPG)-derived right hand PRV (R-PRV) and left hand PRV (L-PRV) measures in 14 patients over 1 year after coronary artery bypass graft (CABG) surgery.

**Method:** The ECG and PPG signals of the patient were recorded simultaneously for 10 min in a supine position. The last 512 stationary RR intervals (RRI) and peak-to peak intervals (PPI) of pulse wave were derived for data analysis. Bland-Altman plot was used to assess the agreement among HRV and both hand PRV measures, while linear regression analysis was used to examine the relationship among corresponding measures of HRV, R-PRV, and L-PRV.

**Result:** The results revealed significant differences in total power (TP), very low-frequency power (VLF), low-frequency power (LF), high-frequency power (HF), and normalized VLF (VLFnorm) among HRV, R-PRV, and L-PRV. Bland-Altman plot analysis showed good agreements in almost all measures between R-PRV and L-PRV, except insufficient agreement was found in LF/HF. Insufficient agreements were found in root mean square successive difference (RMSSD), normalized HF (HFnorm), and LF/HF indices between HRV and L-PRV, and in VLFnorm, HFnorm, and LF/HF indices between HRV and R-PRV. Linear regression analysis showed that the HRV, R-PRV, and L-PRV measures were all highly correlated with one another (*r* = 0.94 ~ 1; *p* < 0.001).

**Conclusion:** Though PRV measures of either hand are not surrogates of HRV measures, they might still be used to evaluate the autonomic nervous functions of CABG patients due to the moderate to good agreements in most time-domain and frequency-domain HRV measures and the strong and positive correlations among HRV and both hands PRV measures in CABG patients.

## Introduction

Heart rate (HR) variability (HRV) refers to the fluctuation of HR responses around the mean HR. The underlying mechanisms to modulate cardiac-related activation are related to autonomic nervous activities and other physiological system regulations (1–3). The temporal and spectral components of HRV can be used to identify the sympathovagal interaction in various pathophysiological conditions, such as acute myocardial infarction (4, 5), prediction of morbidity and mortality (6), identification of septic patients in the intensive care unit (7), and prediction of severity for septic patients in the emergency department (8).

In clinical practice, the health practitioners frequently use palpation technique to determine the pulses rate (PR) of the patients. Recently, wearable devices are frequently used to facilitate PR evaluation for health monitoring. For example, smartwatches and smartphones with built-in photoplethysmographic (PPG) sensors have been extensively used to evaluate the daily change in cardiovascular responses (9). These biomarkers can be further applied to clinical diagnosis, e-health management, and exercise adaptation (10). Thus, PPG assessment of pulse provides convenient and friendly facilitation to monitor cardiovascular health in general and clinical populations.

The PPG detection from different body regions has been reported in recent HRV and PR variability (PRV) studies (11, 12). However, this alternative use of HRV and PRV to assess cardiac-related health is controversial. Previous studies comparing measures between blood pressure waveforms and HRV demonstrated that both methods were reliable to assess cardio-related changes in sympathovagal interaction (13, 14). In a clinical study, moderate to good agreement between HRV and PRV during 1 min deep breath controlled at 6 times per minute and a standard 5 min short-term record has been reported in clinical patients with gynecological and pain medicine practice (15). Conversely, a discrepancy between PRV and HRV measures has been reported in cold exposure (11), spectral analysis (16), during obstructive sleep apnea events (17), and healthy subjects (18). The discrepancy between HRV and PRV measures is related to the blood contents and the structure of the radial artery on the arterial pulse wave propagation (19). The difference in experimental conditions might also play a role, such as ambient temperature (20), respiratory control (21), body position (22).

Pathological studies have shown that patients after coronary artery bypass graft surgery (CABG) have significantly reduced sensitivity in HRV modulation (23). Thus, the HRV assessment can be used to do risk stratification and to monitor the recovery of cardiac health after CABG surgery. However, conventional ECG recording may not be obtainable in CABG patients during home-based recovery. Since PPG technology via smartphone and wearable devices has been widely used nowadays to monitor HRV and PRV, the PRV measures of either hand may be the alternative method to monitor the autonomic nervous function in patients with cardiovascular diseases (24).

This study aimed to investigate (1) the limits of agreement between ECG-derived HRV and both hands PPG-derived PRV measures in patients after CABG surgery; (2) the correlations among measures of HRV and both hands PRV in CABG patients.

## Materials and Methods

### Subjects

Fourteen patients after CABG surgery over 1 year were recruited in this study. All patients were requested to refrain from alcohol or caffeine ingestion 24 h prior to participation in the study. Exclusion criteria included atrial fibrillation, frequent premature ectopic complexes, the use of class I antiarrhythmic medication, and myocardial infarction within the last 6 months. This study has been approved by the Institute Review Board of Taipei Veterans General Hospital. The experimental procedures were introduced to the patients, and written informed consents were obtained prior to the study. This study was undertaken in accordance with the Declaration of Helsinki.

### Heart Rate and Pulse Rate Variabilities

After 5 min rest in a supine position, the ECG and PPG signals of the patient were recorded simultaneously using the PowerLab 16sp with 16 channels (ML795 PowerLab/16SP, ADInstruments, Sydney, Australia) for 10 min. Three self-adhesive ECG electrodes were placed onto the chest parallel to the longitudinal heart axis for ECG recording. The pulse wave signals were recorded at the index fingertip of both hands via infrared PPG probes (MLT1020; ADInstruments, CO Springs, CO, USA). A custom-written program was used to collect ECG and PPG signals (MathWorks Inc., Natick, Massachusetts, USA). An analog-to-digital converter with a sampling rate of 400 Hz was set for data acquisition.

A peak detection algorithm was developed to detect the peaks of the R waves in the QRS complexes in the ECG tracing using a wavelet-based method along with multiscale differential operator (25). The length of the interval between successive peaks of R waves in the QRS complexes was defined as the RR interval (RRI) of that pair of R waves. The highest peak of the pulse wave following the R wave in the QRS complex was detected using a similar peak detection algorithm. The length of the interval between successive peaks of pulse waves in the PPG tracing was defined as the peak-to-peak intervals (PPI) of pulse waves. The last 512 stationary RRI and PPI were used for subsequent data analysis.

Both time-domain and frequency-domain measures of HRV and both hands PRV were compared. Time-domain measures included mean RRI (Mn), heart rate (HR), standard deviation and root mean square successive difference (RMSSD) of RRI or PPI (SDNN), and coefficient of variation of RRI or PPI (CVNN = SDNN/Mn). Frequency-domain measures were the individual powers in the power spectra of HRV and PRV. The power spectra of RRI and PPI were analyzed via fast Fourier transformation (FFT). Direct current components were excluded before computing the powers of individual frequency bands in the power spectra using FFT. The area-under-the-curve of the spectral peaks within the range of 0.01–0.4, 0.01–0.04, 0.04–0.15, and 0.15–0.4 Hz were calculated as the total power (TP), very low-frequency power (VLF), low-frequency power (LF), and high-frequency power (HF), respectively. The normalized high-frequency power (HFnorm = HF/TP) was used as the index of vagal modulation (26); the normalized low-frequency power (LFnorm = LF/TP) as the index of sympathetic and vagal modulation (27); and the low-/high-frequency power ratio (LF/HF) as the index of sympathovagal balance. The very low-frequency power (VLF) was used as the index of renin-angiotensin-aldosterone system and vagal withdrawal (28, 29).

In FFT, the best known use of the Cooley–Tukey algorithm is to divide the transform into two pieces of size *n*/2 at each step. The number of samples used in the FFT is therefore limited to power-of-two sizes, though any factorization can be used in general. Therefore, a sample size of 2^n^ is often used in FFT. In this study, 2^9^ = 512 RRI were used so that the ECG and PPG recording time can be restricted to within 10 min if the heart rate of the study subject is not <52 beats per minute. A long recording time of ECG and PPG might result in instability in the ECG and PPG tracing due to drowsiness, agitation, body movement, etc. The RRI and PPI thus obtained might not be stationary anymore.

### Statistical Analyses

Data are presented as the median and interquartile range (IQR, 25 ~ 75%). Variance of different measures among HRV, and both hand PRV were compared using Friedman repeated-measures analysis of variance (ANOVA) on ranks. All pairwise comparisons were further processed using the Tukey test. Additionally, the agreement between the HRV and PRV measures was assessed using Bland-Altman plots (30). Bias was calculated based on the average value of the difference between measures. The ratio of half difference between upper and lower 95% confidence limits to the mean of all pairwise measurement means (MPM) was calculated. A ratio < 0.1 was defined as good agreement; a ratio between 0.1 and 0.2 was defined as moderate agreement; and a ratio >0.2 was defined as insufficient agreement (31). Linear regression analysis was performed to determine the relationship between common measures of HRV and both hands PRV. All statistical analyses were performed using SigmaPlot 13 software (SPSS Inc., Chicago, IL, USA).

## Results

### Demographics and Clinical Profiles

Fourteen patients recruited in this study had a mean age of 59.5 years, and 9 (64.3%) of them were male. The demographics, clinical profiles, and current medication are presented in Table 1.

**Table 1:** Demographics and clinical characteristics of the patients receiving CABG.

**Age (yrs)**	**63.5 (55.5 ~ 66.3)**
**Gender**	
**Male**	**9 (64.3)**
**Female**	**5 (35.7)**
**Body height (cm)**	**162 (157 ~ 168)**
**Body weight (kg)**	**64.1 (57.3 ~ 80.0)**
**Body mass index (kg/m^2^)**	**25.1 (22.1 ~ 27.0)**
**History**	
**Previous myocardial infarction**	**5 (35.7)**
**Hypertension**	**11 (78.6)**
**Diabetes mellitus**	**6 (42.9)**
**Hyperlipidemia**	**4 (28.6)**
**Medication**	
**Beta-Blocker**	**5 (35.7)**
**Calcium antagonist**	**9 (64.3)**
**Nitrates**	**12 (85.7)**
**Angiotensin-Converting enzyme inhibitor**	**6 (42.9)**
**Digitalis**	**2 (14.3)**
**Aspirin**	**10 (71.4)**

*Data are presented as median and interquartile range (IQR, 25 ~ 75%) or number (percentage). CABG, coronary artery bypass graft surgery*.

### Comparisons Among HRV, Left Hand PRV, and Right Hand PRV

The results revealed significant differences in TP, VLF, LF, HF, and VLFnorm among HRV, right hand PRV (R-PRV), and left hand PRV (L-PRV) (Table 2). Pairwise comparisons showed that the VLF, HF, and VLFnorm of R-PRV were significantly greater than those of HRV, whereas the LF of L-PRV was significantly greater than that of HRV.

**Table 2:** Comparisons among HRV, L-PRV, and R-PRV measures in patients after CABG.

**Parameters**	**HRV**		**L-PRV**		**R-PRV**		
	**Median (IQR)**	**CV (%)**	**Median (IQR)**	**CV (%)**	**Median (IQR)**	**CV (%)**	***P*** **-value**
**Time-domain variables**							
Mn (ms)	783.7 (747.9 ~ 843.6)	8.1	783.7 (747.9 ~ 843.6)	8.1	783.7 (747.9 ~ 843.6)	8.1	0.779
SDNN (ms)	28.6 (19.4 ~ 35.9)	59.2	28.5 (18.3 ~ 36.1)	58.1	28.6 (18.4 ~ 36.0)	58.3	0.863
CVNN (%)	0.03 (0.02 ~ 0.04)	53.5	0.03 (0.02 ~ 0.05)	52.4	0.03 (0.02 ~ 0.05)	52.3	0.223
RMSSD (ms)	18.1 (11.2 ~ 46.4)	92.0	21.9 (10.6 ~ 45.9)	89.1	20.9 (10.7 ~ 45.4)	90.1	0.865
**Frequency-domain variables**							
TP (ms^2^)	271.2 (92.5 ~ 467.6)	139.7	277.4 (95.0 ~ 511.1)	138.0	278.9 (98.8 ~ 516.6)	138.1	0.033#
VLF (ms^2^)	66.9 (34.2 ~ 83.5)	81.0	66.9 (34.5 ~ 83.6)	81.7	67.6 (35.4 ~ 84.1)*	81.2	0.024#
LF (ms^2^)	56.6 (28.2 ~ 198.3)	138.1	59.5 (30.3 ~ 196.6)*	138.6	57.5 (33.5 ~ 196.1)	139.0	0.011#
HF (ms^2^)	40.6 (16.2 ~ 335.5)	173.8	62.2 (20.4 ~ 336.7)	170.1	61.3 (20.9 ~ 331.9)*	170.1	0.042#
VLFnorm (nu)	30.6 (11.5 ~ 48.3)	70.4	33.1 (11.6 ~ 47.4)	68.1	32.6 (11.8 ~ 45.9)*	68.2	0.030#
LFnorm (nu)	27.9 (19.4 ~ 37.3)	60.7	29.1 (18.8 ~ 36.3)	59.8	29.3 (18.2 ~ 35.6)	60.9	0.807
HFnorm (nu)	32.1 (12.5 ~ 61.9)	65.7	30.6 (17.0 ~ 61.5)	61.1	31.6 (19.2 ~ 61.5)	60.4	0.257
LF/HF	0.7 (0.4 ~ 2.7)	127.1	0.8 (0.5 ~ 2.2)	131.8	0.8 (0.4 ~ 1.9)	140.7	0.318

*Data are presented as median (interquartile range, IQR: 25 ~75%). CABG, coronary bypass graft surgery; HRV, heart rate variability; PRV, pulse rate variability; L-PRV, left hand PRV; R-PRV, right hand PRV; CV, coefficient of variation; Mn, mean RR interval; SDNN, standard deviation of RR intervals; CVNN, coefficient of variation of RR intervals; RMSSD, root mean square of successive difference; TP, total power; VLF, very low-frequency power; LF, low-frequency power; HF, high-frequency power; VLFnorm, normalized VLF; LFnorm, normalized LF; HFnorm, normalized HF; LF/HF, low-/high- frequency power ratio; bpm, beats per minute; ms, millisecond; nu., normalized unit*.

* *Significant difference vs. HRV*.

# *Significant difference in group comparison*.

### Bland-Altman Analysis

**Figure 1:**
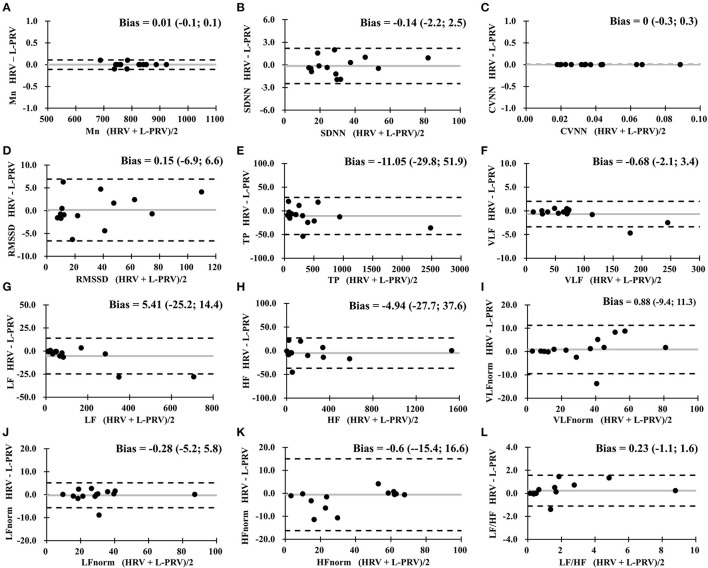
Bland-Altman analysis between HRV measures and L-PRV measures. Gray lines indicate the bias between the measures. **(A)** Mn, mean RR interval; **(B)** SDNN, standard deviation of normal RR intervals; **(C)** CVNN, coefficient of variation of normal RR intervals; **(D)** RMSSD, root mean square of successive difference; **(E)** TP, total power; **(F)** VLF, very low-frequency power; **(G)** LF, low-frequency power; **(H)** HF, high-frequency power; **(I)** VLFnorm, normalized VLF; **(J)** LFnorm, normalized LF; **(K)** HFnorm, normalized HF; **(L)** LF/HF, low-/high- frequency power ratio; L-PRV, left hand pulse rate variability.

Table 3 shows the results of Bland-Altman analysis among HRV, L-PRV, and R-PRV. Good agreements were observed in Mn, SDNN, CVNN, TP, VLF, and VLFnorm; moderate agreements were found in LF, HF, and LFnorm; while insufficient agreements were observed in RMSSD, HFnorm, and LF/HF between HRV and L-PRV (Figure 1).

**Table 3:** Bland-Altman analysis of measuring variables among HRV, L-PRV, and R-PRV in patients after CABG.

**Parameters**	**HRV vs. L-PRV**		**HRV vs. R-PRV**		**R-PRV vs. L-PRV**				
	**MPM**	**Ratio**	**Agreement**	**MPM**	**Ratio**	**Agreement**	**MPM**	**Ratio**	**Agreement**
Mn (ms)	797.2, 64.7	1.39 × 10^−4^	Good	797.2, 64.7	1.19 × 10^−4^	Good	797.2, 64.7	6.71 × 10^−5^	Good
SDNN (ms)	31.7, 18.6	0.076	Good	31.6, 18.6	0.063	Good	31.7, 18.4	0.035	Good
CVNN (%)	3.9, 2.1	0.076	Good	3.9, 2.1	0.065	Good	3.9, 2.1	0.037	Good
RMSSD (ms)	34, 30.7	0.203	Insufficient	33.9, 30.8	0.170	Moderate	33.8, 30.2	0.082	Good
TP (ms^2^)	455.9, 632.9	0.092	Good	455.7, 633	0.088	Good	461.3, 636.8	0.022	Good
VLF (ms^2^)	78.2, 63.6	0.035	Good	78.3, 63.5	0.030	Good	78.6, 64	0.012	Good
LF (ms^2^)	139.6, 193.0	0.142	Moderate	139.3, 192.9	0.135	Moderate	142, 197	0.023	Good
HF (ms^2^)	238.2, 409.3	0.137	Moderate	238.2, 409.5	0.128	Moderate	240.7, 409.4	0.036	Good
VLFnorm (nu)	35.9, 22.3	0.026	Good	35.8, 22.3	0.268	Insufficient	70.7, 43.4	0.017	Good
LFnorm (nu)	30.8, 18.5	0.179	Moderate	30.7, 18.6	0.168	Moderate	30.9, 18.6	0.039	Good
HFnorm (nu)	36.8, 23	0.434	Insufficient	37, 23	0.40	Insufficient	37.3, 22.6	0.055	Good
LF/HF	1.8, 2.4	0.739	Insufficient	1.9, 2.5	0.689	Insufficient	1.7, 2.4	0.275	Insufficient

*MPM is presented as mean and standard deviation, and ratio as 0.5 × (range of LA)/MPM. CABG, coronary bypass graft surgery; HRV, heart rate variability; PRV, pulse rate variability; MPM, mean of pairwise means. Ratio = 0.5 (range of LA)/MPM. Mn, mean RR interval; SDNN, standard deviation of RR intervals; CVNN, coefficient of variation of RR intervals; RMSSD, root mean square of successive difference; TP, total power; VLF, very low-frequency power; LF, low-frequency power; HF, high-frequency power; VLFnorm, normalized VLF; LFnorm, normalized LF; HFnorm, normalized HF; LF/HF, low-/high- frequency power ratio; bpm, beats per minute; ms, millisecond; nu, normalized unit*.

**Figure 2:**
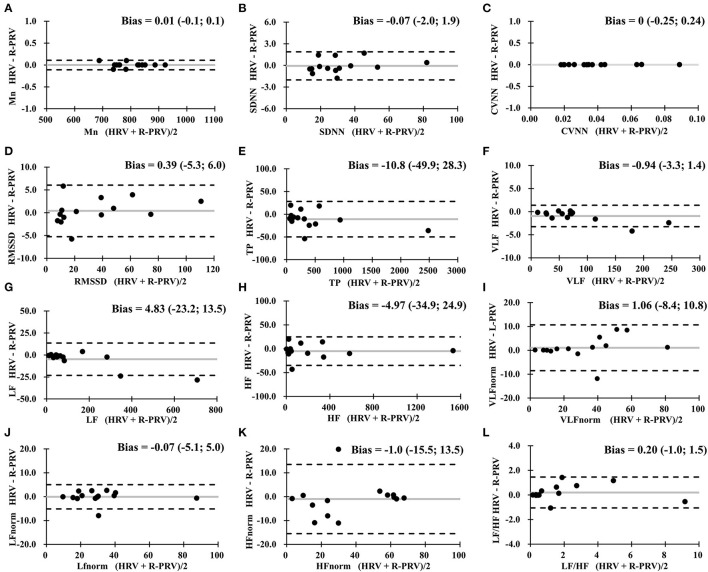
Bland-Altman analysis between HRV measures and R-PRV measures. Gray lines indicate the bias between the measures. **(A)** Mn, mean RR interval; **(B)** SDNN, standard deviation of normal RR intervals; **(C)** CVNN, coefficient of variation of normal RR intervals; **(D)** RMSSD, root mean square of successive difference; **(E)** TP, total power; **(F)** VLF, very low-frequency power; **(G)** LF, low-frequency power; **(H)** HF, high-frequency power; **(I)** VLFnorm, normalized VLF; **(J)** LFnorm, normalized LF; **(K)** HFnorm, normalized HF; **(L)** LF/HF, low-/high- frequency power ratio; R-PRV, right hand pulse rate variability.

The comparison between HRV and R-PRV revealed good agreements in Mn, SDNN, CVNN, TP, and VLF; moderate agreement in RMSSD, LF, HF, and LFnorm; and insufficient agreement in VLFnorm, HFnorm, and LF/HF between HRV and R-PRV (Figure 2).

**Figure 3:**
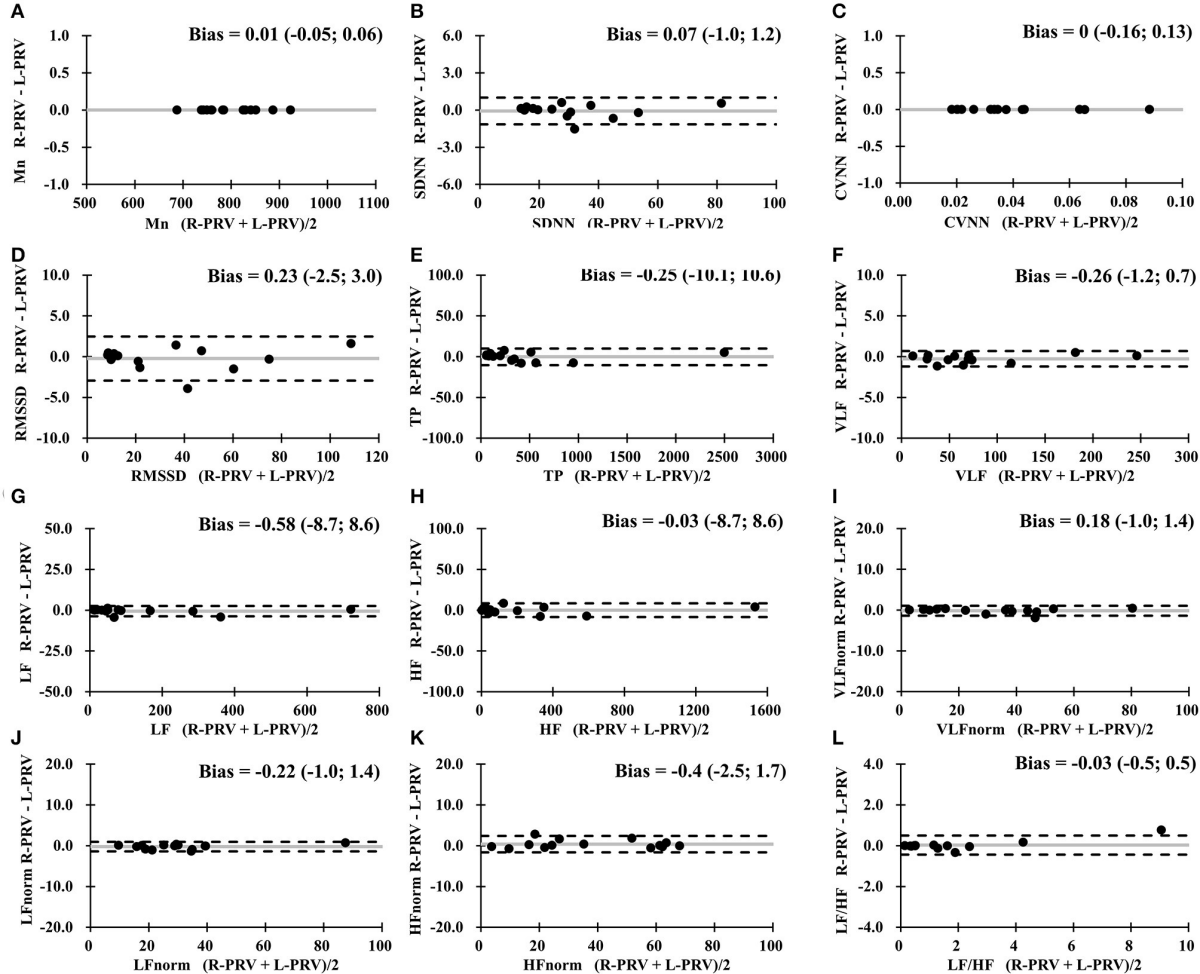
Bland-Altman analysis between R-PRV measures and L-PRV measures. Gray lines indicate the bias between the measures. **(A)** Mn, mean RR interval; **(B)** SDNN, standard deviation of normal RR intervals; **(C)** CVNN, coefficient of variation of normal RR intervals; **(D)** RMSSD, root mean square of successive difference; **(E)** TP, total power; **(F)** VLF, very low-frequency power; **(G)** LF, low-frequency power; **(H)** HF, high-frequency power; **(I)** VLFnorm, normalized VLF; **(J)** LFnorm, normalized LF; **(K)** HFnorm, normalized HF; **(L)** LF/HF, low-/high- frequency power ratio; L-PRV, left hand pulse rate variability; R-PRV, right hand pulse rate variability.

In the comparison between the measures of R-PRV and L-PRV, good agreements were observed in almost all measures, except for insufficient agreement found in LF/HF (Figure 3).

### Linear Regression Analysis

**Figure 4:**
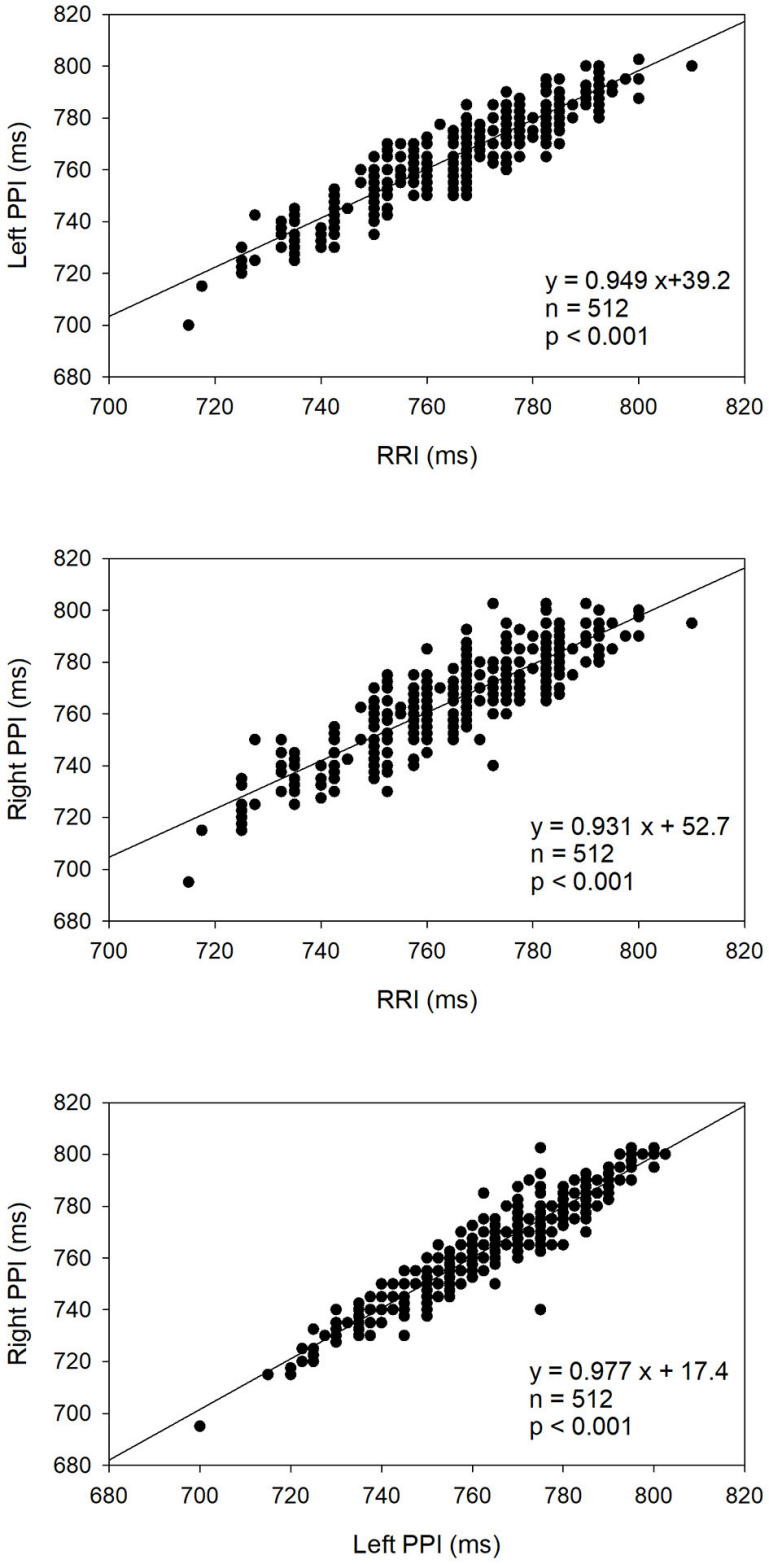
Linear correlation analysis among the RRI, left hand PPI, and right hand PPI in a representative patient.

Figure 4 shows the linear correlations among the RRI, left hand PPI, and right hand PPI in a representative patient. There are very significant and strong positive correlations among RRI and both hands PPI in that study subject, indicating that both hand PPI is associated strongly and positively with the RRI. As demonstrated in Table 4, there were significant and strong positive correlations among all measures of HRV, R-PRV, and L-PRV (*r* ranged from 0.943 to 1, *p* < 0.01).

**Table 4:** Linear regression analysis among HRV, L-PRV, and R-PRV measures in patients after CABG.

**Parameters**	**HRV vs. L-PRV**	**HRV vs. R-PRV**	**L-PRV vs. R-PRV**
Mn (ms)	1 < 0.01	1 < 0.01	1 < 0.01
SDNN (ms)	0.998 < 0.01	0.999 < 0.01	1 < 0.01
CVNN (%)	0.998 < 0.01	0.998 < 0.01	0.999 < 0.01
RMSSD (ms)	0.994 < 0.01	0.996 < 0.01	0.999 < 0.01
TP (ms^2^)	1 < 0.01	1 < 0.01	1 < 0.01
VLF (ms^2^)	1 < 0.01	1 < 0.01	1 < 0.01
LF (ms^2^)	1 < 0.01	1 < 0.01	1 < 0.01
HF (ms^2^)	0.999 < 0.01	0.999 < 0.01	1 < 0.01
VLFnorm (nu)	0.974 < 0.01	0.978 < 0.01	1 < 0.01
LFnorm (nu)	0.989 < 0.01	0.990 < 0.01	1 < 0.01
HFnorm (nu)	0.943 < 0.01	0.951 < 0.01	0.999 < 0.01
LF/HF	0.963 < 0.01	0.967 < 0.01	0.998 < 0.01

*Data are presented as r and p-values. CABG, coronary bypass graft surgery; HRV, heart rate variability; PRV, pulse rate variability; L-PRV, left hand PRV; R-PRV, right hand PRV; Mn, mean RR interval; SDNN, standard deviation of RR intervals; CVNN, coefficient of variation of RR intervals; RMSSD, root mean square of successive difference; TP, total power; VLF, very low-frequency power; LF, low-frequency power; HF, high-frequency power; VLFnorm, normalized VLF; LFnorm, normalized LF; HFnorm, normalized HF; LF/HF, low-/high- frequency power ratio; bpm, beats per minute; ms, millisecond; ms^2^, millisecond squared; nu, normalized unit*.

## Discussion

This study investigated the agreement and correlation of time-domain and frequency-domain HRV indices between ECG-derived HRV and finger PRV (right and left hands) in CABG patients after 1 year of surgery. The primary finding was that both hands PRV cannot be used as the surrogate of HRV as evidenced by (1) insufficient agreement in RMSSD, HFnorm, and LF/HF between HRV and L-PRV, and (2) insufficient agreement in VLFnorm, HFnorm, and LF/HF between HRV and R-PRV. Clearly, there was insufficient agreement in LF/HF between HRV measures and PRV measures of either hand. The secondary finding was that both hands PRV measures have a near perfect correlations with HRV measures, indicating that both hand PRV measures can also be used to evaluate autonomic nervous modulation in CABG patients. If the latter finding is true, then the PRV of either hand can be used as a user-friendly and low-cost (i.e., smartphone and smartwatch) option for the regular evaluation and monitoring of autonomic nervous function in CABG patients and possibly in patients with other cardiovascular diseases.

In this study, the Friedman test revealed significant differences in TP, VLF, LF, HF, and VLFnorm among HRV, R-PRV, and L-PRV. Overestimation of HRV variables in VLF, LF, HF, and VLFnorm of R-PRV was observed when PRV was used to compare to HRV. It seems such observation only occurred in frequency-domain HRV indices.

The limits of agreements were found to be of a moderate to good levels in Mn, SDNN, CVNN, TP, VLF, LF, HF, and LFnorm, while an insufficient agreement was found in RMSSD, VLFnorm, HFnorm, and LF/HF between HRV and both hand PRV. Our previous study supported such findings where a poor agreement was found between both hand PRV and ECG-derived HRV in healthy adults (18). Furthermore, pathological conditions such as obstructive sleep apnea (17) and blood pressure hypertension or hypotension (32) could potentially lead to a large measurement bias between the PRV and HRV. Conversely, moderate to good agreements between HRV and PRV have been reported in 343 clinical patients with gynecological and pain medicine practice during deep breath and normal breath conditions (15). The controversial findings may be related to methodological considerations (signal processing, identification of fiducial points, sample rate etc…) and physiological conditions (arterial vessel, respiratory activity, recording site etc…) among the studies (12).

In term of the time-domain HRV indices, RMSSD showed insufficient agreement between HRV and PRV of either hand. In HRV measures, the RMSSD is a strong indicator of vagal tone (33) and is considered a primary biomarker to identify autonomic adaptation in responses to psychological (34) and physiological stimuli (35). It was assumed that pathological conditions could play a role in affecting the limits of agreement between PRV and HRV. Mejía-Mejía et al. (32) showed that hospitalized patients in an intensive care unit have the largest bias error in RMSSD between PRV and HRV measures, compared to others time-domain indices such as SDNN, RRI, and pNN50. Furthermore, Khandoker et al. (17) reported a significant difference in RMSSD when PRV and HRV were recorded during 2 min obstructive sleep apnea events. The inaccuracy measures of RMSSD between PRV and HRV was also identified in healthy adults (18). The poor accuracy of measures between PRV and HRV may be related to the association of mathematical calculation and fiducial points of measures.

Interestingly, large measurement errors were observed in HFnorm and LF/HF when right or left hand PRV was compared to the ECG-derived HRV. These two HRV variables provide an essential view to understanding the vagal activation and sympathovagal balance in health status (33). The high-frequency component of HRV is known to be caused by respiration. The effect of respiration on the variation in RRI and PPI might be different because of the intervening radial artery. The time for the pulse wave to travel from the heart to the index fingertip of either hand through the radial artery might be affected by respiration, leading to a greater effect of respiration on the higher frequency component of PRV. The greater impact of respiration on PPI might be the reason why the lower-frequency components agree better than the higher-frequency components between HRV and PRV. Further explorations of these factors are warranted to validate this speculation.

Although autonomic modulation is similar between PRV and HRV, these two measures are not surrogates of each other due to insifficuint agreement found in RMSSD, HF, and LF/HF. Recent studies provide solid evidence to support the profound effects of cardiac and vascular mechanisms on PPG recording, suggesting distinctive features between HRV and PRV (19). Another factor contributing to the difference between HRV and both hand PRV might be the variation in time used by the blood to travel from the heart to the radial artery. Nevertheless, the accuracy of both hand PRV measures as the surrogate of HRV estimation is not convincing in CABG patients. A potential risk to underestimate/overestimate the HRV values by using either hand PRV should be noted (15). We speculate that measurement error may occur when PRV measures of either hand are used as a surrogate of HRV in CABG patients.

To identify the limits of agreement on ipsilateral hand PRV, the R-PRV, and L-PRV were used for comparison in our study. Previously Wong et al. (18) reported asymmetry in PRV modulation between both hands in healthy seniors, as observed by RMSSD, TP, HF, HFnorm, and LF/HF variables. Conversely, our finding only revealed insufficient agreement in LF/HF between both hands PRV. The difference in the accuracy of hand PRV measures between healthy seniors and CABG patients might be related to the structure of radial artery, the asymmetry of cardiovascular anatomy in the thorax, and the less sensitivity of vagal-related control over arterial modulation after CABG surgery (23). Thus, using hand PRV measure as an independent biomarker to evaluate the overall cardiovascular function in the target population should be considered.

As demonstrated in Table 4, near perfect and perfect correlations were identified in all pairwise comparisons. The results of linear correlation demonstrated a strong link between both hand PRV and HRV measures for the evaluation of autonomic nervous function in CABG patients. In particular, this finding was associated with a similar coefficient of variance in intra-subject comparisons, as shown in Table 2. Our findings were in line with previous reports, which showed significant strong positive correlations between PRV and HRV in healthy adults (11, 18, 34) and in patients with hypoglycemia syndrome (35).

The discrepancy of 5 min short-term records in RMSSD, VLFnorm, HFnorm, and LF/HF variables found in the present study may be influenced by two physiological factors. The first factor is related to vascular determinants present in both hands PRV but not in ECG-derived HRV. The hemodynamic functions are mainly determined by the quality and structure of blood vessels, vascular stiffness after the left ventricle contraction, and the viscosity and osmolarity of the blood. Measuring arterial responses at the fingertips may be potentially influenced by these physiological factors during PPG assessment (12, 19). The second factor is related to the discrepancy in biosignal transmission between ECG and both hands PRV. The information transmitted from the R wave of ECG to the subsequent peak of pulse wave may be affected by respiratory control, stiffness of radial artery, constituents of blood, medication, and multiple chronic diseases. The CABG patients in this study had more than one chronic disease and used many kinds of medication, including cardiovascular medicine. Previous studies examining cardiovascular waveforms in patients with cardiovascular diseases supported this conjecture (36–38).

In CABG, reverse segments of the great saphenous vein or the pedicle graft of the left internal mammary artery were harvested and bridged between the coronary artery distal to the stenotic lesion and ascending aorta. In this study, all patients received CABG surgery with the graft taken from their internal mammary artery or great saphenous vein. None of them received grafts from their radial arteries. Therefore, the quality of PPG signals taken from the radial arteries of both hands were not affected by the CABG surgery in this study.

The practical implication of the current study highlights the feasibility of using PRV for interpreting cardiac health in CABG patients. The advancement of PRV recordings is the result of the widespread use of built-in PPG sensors (i.e., smartphone, smartwatch, or pulse oximeter etc.). The PRV recorded from the fingertip is easily assessable and convenient as a daily routine (10). This routine process may be used as a diagnostic tool to reduce the mortality rate of coronary events or a clinical evaluation for postoperative care (39). Recently, a clinical study demonstrated that the high quality of smartphone-based PPG recordings provided a similar level of sensitivity and accuracy in diagnosing atrial fibrillation by physicians (40). Thus, future studies are recommended to use PRV signals to identify subsequent changes in cardiovascular functions in patients after CABG surgery.

This study has several limitations. Firstly, more patients after CABG surgery are needed to validate our findings in this small-scale study. Secondly, the extension of the findings of this study to patients with other kinds of cardiovascular disease needs further evidence to verify as only CABG patients were recruited in the current study. Thirdly, only post-surgical HRV and PRV measures in the CABG patients were taken over a 1 year period. The results of this study may not be applicable to patients during the recovery phase after CABG surgery in the hospital setting and during home-based recovery phase within a year. Future studies should compare the accuracy between HRV and both hands PRV measures in other kinds of cardiovascular disease and CABG patients within 1 year after surgery. Fourthly, this study was carried on using short-term spectral HRV/PRV analysis, which is subject to the variation in the physiological and psychological conditions and the medications of the patients. Finally, this study was a cross-sectional investigation. The outcomes of this study are not comparable to longitudinal measures between HRV and PRV in CABG patients. Cautions should be exercised in the interpretation of the experimental data.

## Conclusion

Both hand PRV measures cannot be used as the surrogate of ECG-derived HRV measures in CABG patients due to insufficient agreements in RMSSD, HFnorm, and LF/HF indices which are essential in the evaluation of autonomic nervous function in short-term HRV analysis. The use of PRV measures to monitor cardiac-related health in patients after CABG surgery over 1 year should be done with caution. However, the use of PRV of either hand for the evaluation of autonomic nervous function might be warranted in CABG patients and possibly other kinds of cardiovarscular diseases because of good agreement in most time-domain and frequency-domain HRV measures and the strong positive correlations among HRV and both hand PRV measures in CABG patients.

## Data Availability Statement

The raw data supporting the conclusions of this article will be made available by the authors, without undue reservation.

## Ethics Statement

The studies involving human participants were reviewed and approved by Institute Review Board of Taipei Veterans General Hospital. The patients/participants provided their written informed consent to participate in this study.

## Author Contributions

Y-SC contributed to the study conceptualization, investigation, and writing (including reviewing and editing) of the manuscript. Y-YL contributed to the methodology, data acquisition, statistical analysis, and writing (including reviewing and editing) of the manuscript. C-CS contributed to the surgery, primary care and recruitment of CABG patients, administration, and writing (including reviewing and editing) of the manuscript. C-DK contributed to the study conceptualization, project administration, methodology, supervision, data interpretation, and writing (including reviewing and editing) of the manuscript. All authors contributed to the article and approved the submitted version.

## Funding

This work was supported by a grant VGHTPE93-361-5 from Taipei Veterans General Hospital, and a grant CCMP95-TP-040 from the Committee on Chinese Medicine and Pharmacy, Department of Health, Taipei, Taiwan.

## Conflict of Interest

C-DK is a consultant of the Leadtek Research Inc., New Taipei City, Taiwan. The remaining authors declare that the research was conducted in the absence of any commercial or financial relationships that could be construed as a potential conflict of interest.

## Publisher's Note

All claims expressed in this article are solely those of the authors and do not necessarily represent those of their affiliated organizations, or those of the publisher, the editors and the reviewers. Any product that may be evaluated in this article, or claim that may be made by its manufacturer, is not guaranteed or endorsed by the publisher.
